# Burnout of health care providers during the COVID-19 pandemic: Focus on Medical Oncologists

**DOI:** 10.7150/ijms.54025

**Published:** 2021-03-26

**Authors:** Patrizia Vici, Eriseld Krasniqi, Laura Pizzuti, Gennaro Ciliberto, Marco Mazzotta, Daniele Marinelli, Maddalena Barba

**Affiliations:** 1 Division of Medical Oncology 2, IRCCS Regina Elena National Cancer Institute, Via Elio Chianesi, 00144 Rome, Italy; 2 Scientific Direction, IRCCS Regina Elena National Cancer Institute, Via Elio Chianesi 53, 00144 Rome, Italy; 3 Department of Clinical and Molecular Medicine, "Sapienza" University of Rome, Azienda Ospedaliera Sant'Andrea, 00189 Rome, Italy

**Keywords:** Medical oncologists, burnout, Covid-19, cancer.

## Abstract

The spread of the coronavirus disease 2019 (Covid-19) has challenged hard the national health system worldwide. At any level, the role of health care providers has been rapidly revisited and eventually modified to face the pandemic. The search of the balance between the provision of the most appropriate health-related services and safety of both patients and health care providers has become an indisputable necessity. The consequently increased work load, along with a widespread feeling of intellectual isolation, emotional overload, sense of inadequacy for involvement in tasks and disciplines which are not always familiar have all been proposed as factors related to the onset and/or worsening of the burnout phenomenon. This latter is sadly renown among care givers and is particularly common among medical oncologists. We herein share our perspectives on the burnout phenomenon over the course of the Covid-19 pandemic, with a specific focus on medical oncologists. Results from the most recent and inherent studies are presented and commented in light of hints provided by the experience matured by a quite restricted, still potentially representative, number of professionals figures from the medical oncologists' category. Reasons are proposed to explain the sense of inadequacy currently perceived in relation to the limits imposed by the current pandemic. In more detail, we illustrate the nature and extents of some of the most relevant difficulties in the optimal management of cancer patients and constant efforts towards the scientific upgrade which allows for the improvement of the professional performance. The need for a deeper understanding of the roots and consequences of the Covid-19 pandemic on the mental health of medical oncologists is finally stressed.

The rapid spread of the coronavirus disease 2019 (Covid-19) worldwide has led to the declaration of pandemic on March 11th, 2020 1. The tribute paid this far to the novel infectious disease outbreak in terms of human lives and socio-economic costs frankly crosses the threshold of reasonableness and acceptability.

In the current scenario, the risk of Covid-19 in cancer patients has become a matter of intense debate. In these patients, the most commonly observed immune deficiencies are iatrogenic in nature. As such, their occurrence is most frequently related to surgical procedures, chemotherapy and radiotherapy, either singularly or combined 2. On this basis, it is reasonable hypothesizing that peculiar treatment-related features, as well as patient and disease characteristics, may all concur to alter the host immune competence. Finally, the risk of SARS-CoV-2 infection and disease can be increased, with also less favorable outcomes.

Evidence concerning this topic has flourished rapidly. The quite alarming message conveyed by the very first reports on Covid-19 in cancer patients from China has soon been carefully re-examined in reference to some methodological limitations related to the observational nature of the cited studies, as well as to their limited sample size 3-4. Subsequently, several authors have drawn the scientific community attention on the key influence exerted on the risk of SARS-CoV-2 infection and related disease by demographic characteristics, age and pre-existing co-morbidities, along with the existing differences in terms of restrictions applied at the single nation level following the pandemic declaration 5-8. On this basis, an overall attitude towards a less “definitive” position concerning this topic seems plausible.

It is anyway undeniable, not to say obvious, that decisions concerning cancer patients management in the Covid-19 era cannot be exclusively oriented by current recommendations in clinical practice.

In this context, re-examination of any single clinical decision has thus become an indisputable duty. Oncologists are now called to outline a cost-benefit balance which they are not accustomed to. Particularly for cancer patients in need of diagnostic workup and/or therapy administration, the risk of SARS-CoV-2 infection and disease, which is inevitably increased by frequent access to the health care facilities and reiterated contacts with care providers, must be now carefully weighed against the risk of cancer progression and death related to the lack of timely and appropriate cancer-specific interventions.

A plethora of guidelines on how to adapt the oncology practice to the needs dictated by the current pandemic have been rapidly published, including a global approach to patient management and treatment decision-making 9-11. Such guidelines surely provide a precious support to care givers under these specific circumstances. Still, the additional risks and emergency conditions related to the pandemic outbreak have conferred to cancer disease a dimension of “limited manageability” on behalf of the specialists generally involved. This latter may translate particularly, though not exclusively, for medical oncologists into increased occupational stress, which has been associated with depression, anxiety, and has also been related to the burnout syndrome 12.

According to the International Classification of Disease (ICD), burnout is defined as an “occupational phenomenon”, not a medical condition. It is included within the 11^th^ revision of the ICD among the factors influencing health status or generating reasons for contacting health services. It is conceptualized as a syndrome characterized by the following three features: sense of energy depletion or exhaustion; increased mental distancing from one's job, or negative feelings or cynicism related to one's job; and reduced professional accomplishments 13. Burnout is widely and sadly renown among oncologists. It is mostly ascribed to the daily contact with an “incurable” disease. It usually occurs when work or personal pressures exceed an individual's ability to cope with them, and may manifest as physical and mental responses. Long-term, unaddressed burnout among clinicians can have devastating consequences, including chronic pathologic health conditions. Approximately 40% of all physicians experience some form of burnout, according to national surveys. However, the burnout rate among oncologists is higher, with a number of surveys showing incidence exceeding 50-70% 14-18.

Although not equipped with a solid psycho-emotional background, as medical oncologists, we feel the imperative need to report on the peculiarity of our current working conditions and on its changes. As exemplified by the aforementioned guidelines on the adaptations needed to overcome the ongoing pandemic 9-11, over the past few months, decision making at almost any level of patients' management worldwide has been mostly oriented by a sort of “Expert-opinion-based policy”. In the absence of any reasonable alternative, this latter approach has been intended as a surrogate of the needed evidence on which proactive strategies would have been subsequently defined in the nearest future and applied according to different patients' populations needs. These latter would have been addressed in the full consideration of the local prevalence and time course of the SARS-CoV-2 infection and disease. In a few words, while waiting for the development of more suitable investigational platforms, we have sailed on sight. When coming to medical oncologists, transferring these concepts to cancer patients may be particularly tough. Under the current circumstances, oncologists may find it particularly hard reconciling the potentially lethal nature of cancer disease with the likely higher risk of SARS-CoV-2 infection. It is intuitive that the degree of the difficulties experimented by medical oncologists has been significantly exacerbated by the necessary and rapid switch from an “evidence-based” to an “expert-opinion based” approach. Although a multidisciplinary, individual-patient based approach and availability of the previously cited guidelines 9-11 have helped mitigate the deriving sense of disorientation, the currently available weapons against cancer in the Covid-19 era may still be sensed as significantly far from being adequate.

Additional psychological distress may come from an increasingly escalating workload, and personal fears, concerning both themselves and the members of their families. The consequences of Covid-19 in reference to mental health have been recently addressed by Vindegaard and Bensor in a systematic review including evidence concerning both health care workers and non-health care workers. Overall, according to previously set inclusion criteria, a total number of 43 studies were judged suitable for inclusion. Among them, two studies focused on patients with confirmed SARS-CoV-2 infection, while the remaining 41 addressed the indirect effects of the pandemic in different subpopulations, including patients with preexisting psychiatric disturbs, health care providers and the general public. Patients from the two studies including participants with ascertained infection and/or disease showed a high level of post-traumatic stress symptoms (PTSS) (96.2%) and significantly higher level of depressive symptoms (p=0.016). In patients with preexisting psychiatric disorders, worsening of psychiatric symptoms was reported results from the twenty studies investigating health care givers showed increased depression/depressive symptoms, anxiety, psychological distress and poor sleep quality. In the remaining 19 studies, at the general population level, lower psychological well-being and higher scores of anxiety and depression emerged compared to the pre-Covid-19 time window. Female gender, poor-self-related health and relatives with ascertained infection/disease were all associated with higher risk of psychiatric symptoms and/or low psychological well-being. In their conclusions, the authors remark how further research on this topic may improve treatment, mental health care planning and thus lead to more effective preventive measures in potential subsequent pandemics 19.

We would add on the need to investigate this interesting topic across pre-defined categories of health care workers, including medical oncologists. To this aim, the design and conduct of ad *hoc*, adequately sized studies represents a “*sine qua non”* condition.

Differential traits between the professional role played thus far by the oncologists and its currently revised version deserve further mentioning. In order to reduce the overall number of accesses to the health care facilities by applying selective filtering to the visits scheduled, particularly for ambulatory patients, a relevant number of hours *per day* are devoted to the screening of pre-existing lists. Subsequently, *ad hoc* contacts by emails and telephone calls are required to confirm or postpone each visit. For those confirmed, questionnaires' administration is the next step, with a focus on key symptoms and contacts over the prior two weeks with people from high prevalence zones. Depending on the specific answers and activities programmed at the individual patient level, a varying number of nasopharyngeal swabs may be required, with the patient access being granted and the program confirmed upon negative swab/s. In addition, patients whose access is confirmed are clearly informed about the need to drastically reduce the access of the accompaniers. Recommendations concerning this latter issue go not infrequently unheeded and become a source of discussion at the time of the patient access. For patients whose access is differed, telemedicine offers a particularly viable option. Oncologists had to become quickly familiar with platforms possibly allowing also video and audio support 20. For patients requiring treatment initiation or in course of treatment, delaying or modifying cancer specific therapy to avoid frequent exposure to risk for patients is becoming one of the most common choices, as well switching to oral therapies so that home delivery is planned instead of in-clinic administrations. Nevertheless, these changes have important organizational implications and require well detailed and adequately motivated communication.

In addition, although fully understandable, the almost exclusive focus of research on Covid-related issues has abruptly interrupted the development of prior flourishing research pipelines and slowed down the achievements of goals previously expected within the short- and middle term. Patient enrollment in clinical trials has been dramatically reduced or temporary dismissed 21. Similarly, publication of cancer-related manuscripts including original data has been extremely delayed by the lack of available reviewers. National and international cancer meetings has been/are being cancelled, or proposed in their virtual version and only few web-conference are allowed to somewhat surrogate the much more intellectually stimulating “face to face” discussion among specialists 22. The themes of intellectual isolation, emotional overload, sense of inadequacy for involvement in tasks and disciplines which are not familiar have all been recently addressed by Wu and colleagues, who reported on the results of a survey comparing the burnout frequency between a group of oncologists from the Hubei Cancer Hospital in Wuhan, who worked in the Covid-19 frontline, and their colleagues from the same institution, who remained in their usual wards Among the 190 oncologists participating in this survey, the occurrence of burnout was less frequent in the frontline group compared with the usual ward group (13% vs 39%; *p*<0.001). Moreover, a scarce sense of personal accomplishment was less common in the frontline versus the usual ward group (39% vs 61%; *p*=0.002). This latter subgroup declared to be more worried about the infection for themselves and families. These unexpected results may have multiple explanations. First, in frontline physicians, greater awareness concerning the ongoing situation and its evolving may come from being closer to key and relevant decision making nodal points. This translates into more easily available accurate information, and deeper and updated knowledge concerning the new policies and procedures. Conversely, oncologists from the usual wards are conscious of cancer patients vulnerability to infections, tendentially discouraged by the frequent discontinuation of treatments determined by the pandemic, as well as by the delays in follow up visits. This all contributes to a profound distortion of their usual work. Conversely, frontline physicians had direct contact with the results of their care for infected patients, which was more rewarding 23.

In conclusion, a radical reconsideration of the role of medical oncologists in clinical practice is ongoing worldwide due to the pandemic. The burnout phenomenon, and, more generally, the mental health consequences of the work pressure and excessive load in health care providers and, more specifically, in medical oncologists, have now been enriched by additional and significant features, which deserve full consideration for their potential implications on the oncologists performance, both as human beings and professionals figures.
